# Shunt dysfunction and mortality after transjugular intrahepatic portosystemic shunt (TIPS) in patients with portal hypertension

**DOI:** 10.1186/s13244-024-01768-8

**Published:** 2024-08-07

**Authors:** Laura Büttner, Lisa Pick, Martin Jonczyk, Uli Fehrenbach, Federico Collettini, Timo Alexander Auer, Dirk Schnapauff, Maximilian De Bucourt, Gero Wieners, Bernhard Gebauer, Annette Aigner, Georg Böning

**Affiliations:** 1 Charité—Universitätsmedizin Berlin, Corporate Member of Freie Universität Berlin and Humboldt-Universität zu Berlin, Department of Radiology, Charitéplatz 1, 10117 Berlin, Germany; 2 Berlin Institute of Health at Charité – Universitätsmedizin Berlin, Charitéplatz 1, 10117 Berlin, Germany; 3 Charité—Universitätsmedizin Berlin, Corporate Member of Freie Universität Berlin, Humboldt-Universität zu Berlin, Institute of Biometry and Clinical Epidemiology, Charitéplatz 1, 10117 Berlin, Germany

**Keywords:** Transjugular intrahepatic portosystemic shunt, Transjugular intrahepatic portosystemic shunt dysfunction, Stent thrombosis, Stent stenosis, Mortality

## Abstract

**Objectives:**

Transjugular intrahepatic portosystemic shunt (TIPS) is a catheter-based, minimally invasive procedure to reduce portal hypertension. The aim of the study was to investigate dysfunction and mortality after TIPS and to identify factors associated with these events.

**Methods:**

A retrospective analysis of 834 patients undergoing TIPS implantation in a single center from 1993–2018 was performed. Cumulative incidence curves were estimated, and frailty models were used to assess associations between potentially influential variables and time to dysfunction or death.

**Results:**

1-, 2-, and 5-year mortality rates were 20.9% (confidence interval (CI) 17.7–24.1), 22.5% (CI 19.1–25.8), and 25.0% (CI: 21.1–28.8), 1-year, 2-year, and 5-year dysfunction rates were 28.4% (CI 24.6–32.3), 38.9% (CI 34.5–43.3), and 52.4% (CI 47.2–57.6). The use of covered stents is a protective factor regarding TIPS dysfunction (hazard ratio (HR) 0.47, CI 0.33–0.68) but does not play a major role in survival (HR 0.95, CI 0.58–1.56). Risk factors for mortality are rather TIPS in an emergency setting (HR 2.78, CI 1.19–6.50), a previous TIPS dysfunction (HR 2.43, CI 1.28–4.62), and an increased Freiburg score (HR 1.45, CI 0.93–2.28).

**Conclusion:**

The use of covered stents is an important protective factor regarding TIPS dysfunction. Whereas previous TIPS dysfunction, emergency TIPS implantation, and an elevated Freiburg score are associated with increased mortality. Awareness of risk factors could contribute to a better selection of patients who may benefit from a TIPS procedure and improve clinical follow-up with regard to early detection of thrombosis/stenosis.

**Critical relevance statement:**

The use of covered stents reduces the risk of dysfunction after transjugular intrahepatic portosystemic shunt (TIPS). TIPS dysfunction, emergency TIPS placement, and a high Freiburg score are linked to higher mortality rates in TIPS patients.

**Key Points:**

- The risk of dysfunction is higher for uncovered stents compared to covered stents.
- Transjugular intrahepatic portosystemic shunt dysfunction increases the risk of instantaneous death after the intervention.
- A higher Freiburg score increases the rate of death after the intervention.
- Transjugular intrahepatic portosystemic shunt implantations in emergency settings reduce survival rates.

**Graphical Abstract:**

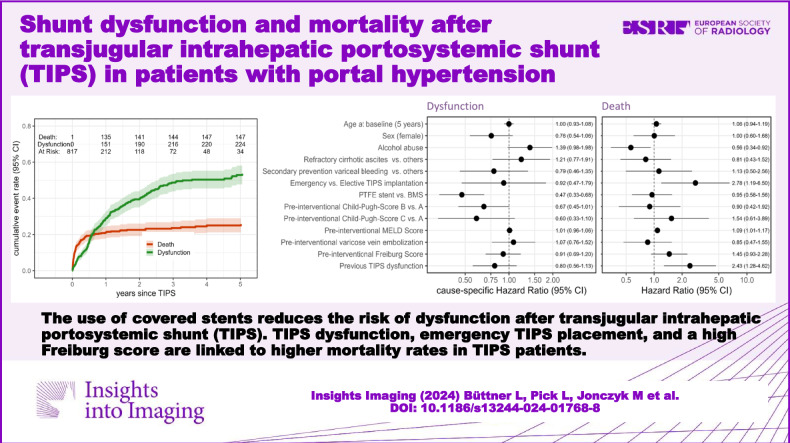

## Introduction

Transjugular intrahepatic portosystemic shunt (TIPS) is a minimally invasive treatment option for patients with portal hypertension [1]. In most cases, portal hypertension is caused by liver cirrhosis; less common causes include Budd-Chiari syndrome, portal vein thrombosis, schistosomiasis, and idiopathic cases [2, 3]. This interventional procedure establishes a transparenchymal connection between the portal vein and the venous system of the liver (portosystemic shunt), reducing pressure in the portal vein and thus the risk of complications of portal hypertension such as ascites or varicose bleeding [1]. Up to 25–50% of patients develop in-stent stenosis with over 50% luminal narrowing within 6–12 months after TIPS when uncovered stents are used, impairing long-term outcomes in these patients [4]. Acute TIPS dysfunction typically occurs in the first weeks after implantation and is due to stent thrombosis. Injury of bile duct structures during the intervention may lead to bile leakage with the development of bile duct fistulas, which also present with acute thrombosis and recurrent occlusion [5]. Delayed dysfunction (months after implantation) is mainly due to pseudointimal hyperplasia or hepatic vein stenosis because of intimal hyperplasia [6]. Post-interventional pseudointimal hyperplasia is mostly caused by the fibrotic healing response to the trauma of shunt creation [4]. An unstented proximal segment of the hepatic vein may lead to intimal hyperplasia and has been described as a predisposing factor for hepatic vein stenosis [7]. Thrombosis and stenosis (bile-related, non-bile-related) are the main mechanisms underlying TIPS dysfunction, while technical reasons (e.g., stent migration) are rare [4, 5]. Other less frequent reasons for TIPS dysfunction are tumor invasion, e.g., in patients with hepatocellular carcinoma (HCC), and hypercoagulopathy, e.g., in patients with Budd-Chiari syndrome [6]. Reports in the literature indicate that uncovered are characterized by a relatively high rate of TIPS dysfunction (50% within 1 year, 80% after 2 years) and with high re-intervention rates [8, 9]. Covered stent grafts have been found to be significantly less likely to develop TIPS dysfunction compared to uncovered stents in randomized controlled trials and meta-analyses [10, 11].

TIPS dysfunction is associated with higher re-intervention and morbidity rates and may even be associated with increased mortality [8, 9]. Awareness of factors potentially relevant to shunt dysfunction and mortality after TIPS implantation may contribute to a better selection of patients who will benefit from TIPS. This may improve both the overall safety of the intervention and patient-specific outcomes. The aim of this study therefore was to identify factors associated with shunt dysfunction and mortality after TIPS implantation.

## Methods

This retrospective study was approved by the responsible ethics committee (EA4/085/17) of our hospital. We included all patients who underwent TIPS implantation at our national center from June 1993 to December 2018. Cases were observed until death, liver transplantation (bridge to transplant), last recanalization attempt (for therapy-refractory TIPS thromboses), iatrogenic TIPS occlusion, or the last patient contact during the observation period (end of observation period December 2018). Recurrent ascites were defined as a clinically relevant increase in abdominal circumference beyond habitual fluctuations. Demographic, blood, clinical, and procedural parameters were recorded. Death was recorded using the electronic medical records stored at our hospital or the medical records of a multicentric cancer registry. TIPS dysfunction was defined as stenosis or thrombosis of TIPS in angiographic TIPS controls. The Freiburg index of post-TIPS survival (FIPS) was established by Bettinger et al for patients with TIPS implantation for treatment-refractory ascites or secondary prophylaxis of variceal bleeding and is based on the parameters age, bilirubin, albumin, and creatinine [12].

Indications for TIPS implantation were established by an interdisciplinary board of hepatologists, radiologists, and surgeons. TIPS was performed as described before [1, 13]. The portosystemic pressure gradient (PSPG) was measured between the portal vein and the inferior vena cava or right atrium [14]. At the beginning of the observation period, PSPG was measured in cmH2O, later the measurement was in mmHg. Measurements in cmH2O were therefore converted to mmHg for comparability. A reduction of the PSPG to 12 mmHg or less was defined as successful TIPS (primary success). In cases where this goal was not accomplished and/or no adequate reduction of clinical symptoms was achieved, re-intervention (e.g., by further dilatation of the tract) was performed to reduce pressure (secondary success). Patients were monitored in the intensive care unit for 24 h after the TIPS intervention before transfer to the normal ward. Follow-up time points after TIPS in our hospital are as follows: day 1 (ultrasound of TIPS flow profile), months 3, 6, and 12 during the first year, then every 12 months. During follow-up visits, the TIPS is visualized sonographically and, if necessary, angiographically.

To describe the study population, we report absolute and relative frequencies for categorical and medians along with interquartile ranges (IQRs) for continuous variables—stratified by both outcome variables, dysfunction, and death. Time to first dysfunction or death is graphically displayed with cumulative incidence plots, along with 95% confidence intervals (CIs). Potentially influential parameters for both outcomes were determined based on previous literature and expert knowledge, such that in the final model the following variables were included: age at TIPS, sex, underlying disease, indication, stent type, pre-interventional Child-Pugh-Score (CPS), model for end-stage liver disease (MELD) score at baseline, varicose vein embolization, Freiburg-score at baseline, and previous TIPS dysfunction (time-dependent). For both outcomes, frailty models with time-dependent covariates were used to account for recurrent events, including a frailty term per patient to account for multiple measurements. Due to death being a competing risk for dysfunction, we estimate cause-specific hazards for the outcome of dysfunction. Based on these models, hazard ratio (HR) and cause-specific HR estimates along with 95% CI were derived. Statistical analyses were performed with R [15], just as additional R packages [16–20].

## Results

**Fig. 1:**
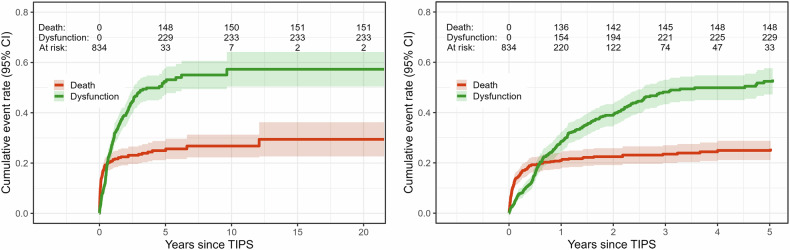
Cumulative event rates for death and dysfunction, along with 95% confidence intervals (CI). Cumulative 1-year, 2-year, and 5-year mortality rates were 21.3%, 22.8%, and 25.3%, respectively (red); cumulative 1-year, 2-year, and 5-year dysfunction rates were 28.7%, 39.1%, and 52.5%

**Fig. 2:**
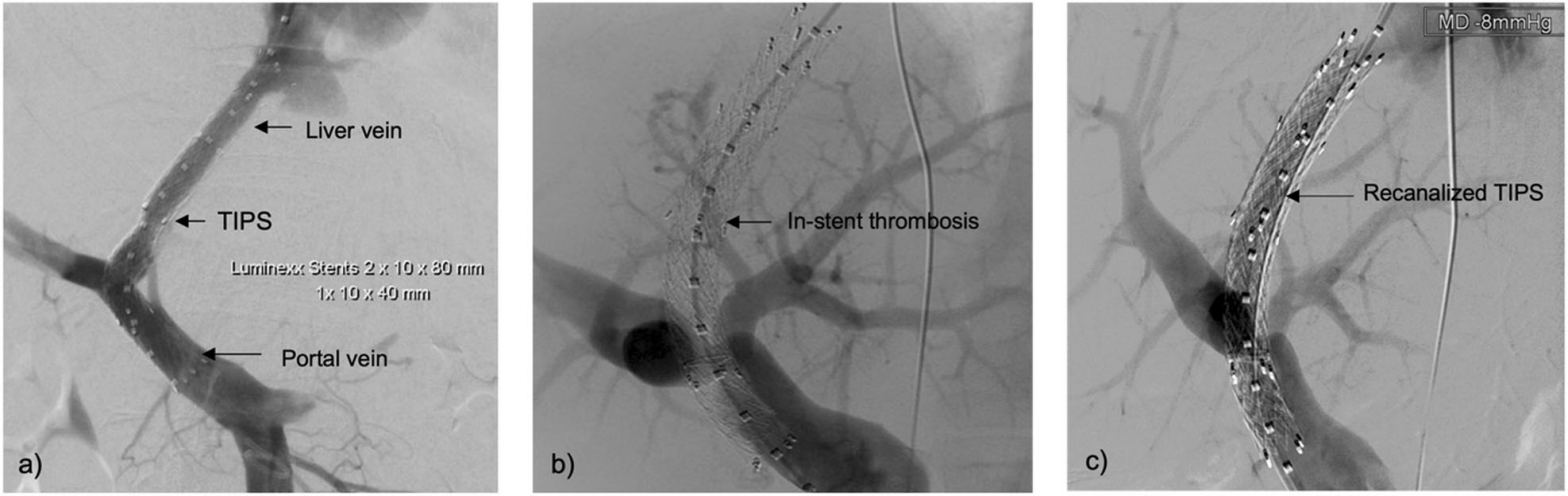
52-year-old female patient with TIPS dysfunction. **a** Digital subtraction angiography (DSA) immediately after TIPS implantation (Luminexx Stents (BD Becton, Dickinson, and Company, 1 Becton Drive Franklin Lakes, NJ 07417-1880, 2 × 10 × 80 mm, 1 × 10 × 40 m) shows TIPS with regular contrast enhancement. **b** DSA after 11 months shows absent contrast enhancement of TIPS. **c** Multiple balloon dilatations using 8 × 40 mm and 10 × 40 mm and 10 × 20 mm Mustang balloons (Boston Scientific Corporation, Marlborough, Massachusetts, USA) (3 bar), followed by dilatation of the proximal TIPS restored flow with a significant pressure reduction of 6 mmHg in the portal system. DSA shows good contrast enhancement of the TIPS with well-preserved perfusion of the intrahepatic portal vein branches. There is a minor constriction between the TIPS stent end and the middle hepatic vein but without hemodynamic relevance. The pressure gradient between the portal vein and the right atrium of 9 mmHg

At least one TIPS dysfunction due to stenosis or thrombosis was observed in 234 out of 562 patients (41.6%), for 272 patients out of the total of 834 patients there was no information available regarding dysfunction. Among those where dysfunction was observed, the first occurred after a median of 7.2 months following the TIPS procedure. 1-year, 2-year, and 5-year dysfunction rates were 28.4% (CI 24.6–32.3), 38.9% (CI 34.5–43.3), and 52.4% (CI 47.2–57.6) (see Fig. 1). An example of TIPS dysfunction due to thrombosis is presented in Fig. 2. All 234 patients with TIPS dysfunction underwent TIPS revision, either by balloon dilatation alone or in combination with stent lengthening or stent-in-stent placement. After TIPS revision, repeated dysfunction occurred in 103 (44.0%) of those patients. Patients with dysfunction had a median age of 56 years and were more often male than female patients (72.6% vs. 27.4%). The most frequent underlying disease in patients with dysfunction as well as in the whole cohort was alcohol abuse (76.8%, *n* = 179); 66.4% (*n* = 372). MELD scores and CPS did only differ slightly between patients with and without TIPS dysfunction. Patients with uncovered stents accounted for 79.9% of TIPS dysfunctions and covered stents for 20.1% (Table 1). Further descriptive results of the study population have been published earlier [21].

**Table 1:** Demographic, clinical, and laboratory parameters data of patients without and with at least one TIPS dysfunction

	No dysfunction^a^ (*n* = 328)	Dysfunction^a^ (*n* = 234)	No death within follow-up (*n* = 637)	Death within follow-up (*n* = 197)	Total (*n* = 834)
Age at baseline (median (IQR))	58.00 (49.00, 64.00)	56.00 (47.00, 64.00)	57.00 (48.00, 64.00)	58.00 (51.00, 65.00)	57.00 (49.00, 64.00)
Sex					
Male	210 (64.0%)	170 (72.6%)	414 (65.0%)	145 (73.6%)	559 (67.0%)
Female	118 (36.0%)	64 (27.4%)	223 (35.0%)	52 (26.4%)	275 (33.0%)
Underlying disease					
Alcohol abuse	193 (59.0%)	179 (76.8%)	435 (68.5%)	117 (59.7%)	552 (66.4%)
Cryptogenic liver cirrhosis	16 (4.9%)	8 (3.4%)	32 (5.0%)	12 (6.1%)	44 (5.3%)
Infectious hepatitis	46 (14.1%)	17 (7.3%)	66 (10.4%)	33 (16.8%)	99 (11.9%)
Other	72 (22.0%)	29 (12.4%)	102 (16.1%)	34 (17.3%)	136 (16.4%)
Missing	1	1	2	1	3
Liver transplant (LT) recipients	13 (4.0%)	4 (1.7%)	15 (2.4%)	12 (6.1%)	27 (3.2%)
Indication					
Refractory cirrhotic ascites	179 (54.6%)	138 (59.0%)	367 (57.6%)	97 (49.2%)	464 (55.6%)
Secondary prevention of variceal bleeding	72 (22.0%)	64 (27.4%)	154 (24.2%)	30 (15.2%)	184 (22.1%)
Others	77 (23.5%)	32 (13.7%)	116 (18.2%)	70 (35.5%)	186 (22.3%)
TIPS implantation setting					
Emergency TIPS implantation	27 (8.2%)	13 (5.6%)	41 (6.4%)	47 (23.9%)	88 (10.6%)
Elective TIPS implantation	301 (91.8%)	221 (94.4%)	596 (93.6%)	150 (76.1%)	746 (89.4%)
Freiburg score (Median (IQR))	−0.09 (−0.64, 0.57)	−0.27 (−0.97, 0.28)	−0.17 (−0.84, 0.38)	0.42 (−0.28, 1.02)	−0.06 (−0.74, 0.56)
Missing	59	54	150	50	200
Comorbidities	94 (28.7%)	51 (21.8%)	165 (25.9%)	59 (29.9%)	224 (26.9%)
Contraindications	36 (11.0%)	15 (6.4%)	62 (9.7%)	24 (12.2%)	86 (10.3%)
Anticoagulation before TIPS	16 (5.7%)	15 (7.0%)	28 (5.0%)	8 (4.5%)	36 (4.9%)
Missing	45	21	77	21	98
Anticoagulation after TIPS	280 (96.2%)	189 (93.1%)	504 (92.0%)	136 (85.5%)	640 (90.5%)
Missing	37	31	89	38	127
Navigation technique					
Sonography	222 (72.3%)	158 (77.1%)	422 (74.7%)	142 (76.3%)	564 (75.1%)
Blind	66 (21.5%)	33 (16.1%)	106 (18.8%)	34 (18.3%)	140 (18.6%)
Other	19 (6.2%)	14 (6.8%)	37 (6.5%)	10 (5.4%)	47 (6.3%)
Missing	21	29	72	11	83
Target liver vein					
Right	264 (89.5%)	179 (88.6%)	489 (88.7%)	167 (92.3%)	656 (89.6%)
Other	31 (10.5%)	23 (11.4%)	62 (11.3%)	14 (7.7%)	76 (10.4%)
Missing	33	32	86	16	102
Stent type					
BMS	178 (59.3%)	155 (79.9%)	368 (67.5%)	123 (70.3%)	491 (68.2%)
PTFE	122 (40.7%)	39 (20.1%)	177 (32.5%)	52 (29.7%)	229 (31.8%)
Missing	28	40	92	22	114
Pre-interventional Child-Pugh-Score					
A	58 (18.1%)	53 (23.3%)	129 (21.0%)	31 (16.4%)	160 (20.0%)
B	202 (62.9%)	150 (66.1%)	397 (64.8%)	98 (51.9%)	495 (61.7%)
C	61 (19.0%)	24 (10.6%)	87 (14.2%)	60 (31.7%)	147 (18.3%)
Missing	7	7	24	8	32
Pre-interventional MELD score (median (IQR))	13.00 (11.00, 18.00)	12.00 (9.00, 16.00)	13.00 (10.00, 16.00)	17.00 (12.00, 21.00)	13.00 (10.00, 18.00)
Missing	33	38	111	34	145
Performance status					
ECOG 0	1 (0.3%)	1 (0.4%)	4 (0.6%)	0 (0.0%)	4 (0.5%)
ECOG 1	47 (14.6%)	42 (18.2%)	101 (16.2%)	7 (3.6%)	108 (13.2%)
ECOG 2	108 (33.4%)	92 (39.8%)	240 (38.5%)	35 (17.8%)	275 (33.5%)
ECOG 3	110 (34.1%)	77 (33.3%)	208 (33.3%)	68 (34.5%)	276 (33.6%)
ECOG 4	57 (17.6%)	19 (8.2%)	71 (11.4%)	87 (44.2%)	158 (19.2%)
Missing	5	3	13	0	13
Pre-interventional Antibiosis	118 (37.2%)	66 (29.5%)	190 (31.0%)	94 (51.9%)	284 (35.8%)
Missing	11	10	25	16	41
Complication	129 (39.4%)	76 (32.6%)	209 (35.8%)	97 (50.8%)	306 (39.5%)
Missing	1	1	53	6	59
Pre-interventional laboratory values					
INR (median (IQR))	1.35 (1.22, 1.54)	1.26 (1.15, 1.41)	1.30 (1.18, 1.49)	1.40 (1.26, 1.61)	1.32 (1.19, 1.51)
Missing	24	22	86	23	109
PTT (s) (median (IQR))	39.50 (36.20, 43.90)	37.75 (34.30, 42.60)	38.90 (35.10, 43.30)	41.30 (36.80, 46.82)	39.30 (35.40, 44.10)
Missing	21	14	56	17	73
Thrombocyts (/nL) (median (IQR))	114.00 (83.00, 175.00)	136.00 (90.00, 196.00)	131.00 (88.00, 189.00)	103.50 (74.25, 160.75)	124.00 (84.50, 183.00)
Missing	15	9	40	15	55
Bilirubin (mg/dL) (median (IQR))	1.40 (0.90, 2.61)	1.12 (0.70, 2.00)	1.21 (0.80, 2.06)	2.13 (1.00, 4.50)	1.40 (0.80, 2.48)
Missing	22	28	83	24	107
Pre-interventional ascites drainage	221 (72.2%)	151 (68.6%)	412 (69.2%)	122 (65.9%)	534 (68.5%)
Missing	22	14	42	12	54
Pre-interventional varicose vein embolization	86 (26.9%)	74 (32.7%)	169 (27.4%)	59 (31.1%)	228 (28.3%)
Missing	8	8	21	7	28

BMS—bare metal stent (uncovered stent). PTFE stent—polytetrafluoroethylene stent (covered stent). ECOG: Eastern Cooperative Oncology Group score: 0—asymptomatic, 1—symptomatic but completely ambulatory, 2—symptomatic, 50% in bed, but not bedbound, 4—bedbound, 5—death [43]. HE: hepatic encephalopathy before TIPS classified according to the West Haven Criteria over the observation period. I: mild symptoms, e.g., loss of sleep and shortened attention span. II: moderate symptoms, e.g., memory loss and slurred speech. III: severe symptoms, e.g., personality changes, confusion, and extreme lethargy. IV: a loss of consciousness and coma [44]

*INR* international normalized ratio, *IQR* interquartile range, *PTFE* polytetrafluoroethylene, *PTT* partial thromboplastin time

^a^ Dysfunction and/or re-dysfunction

**Fig. 3:**
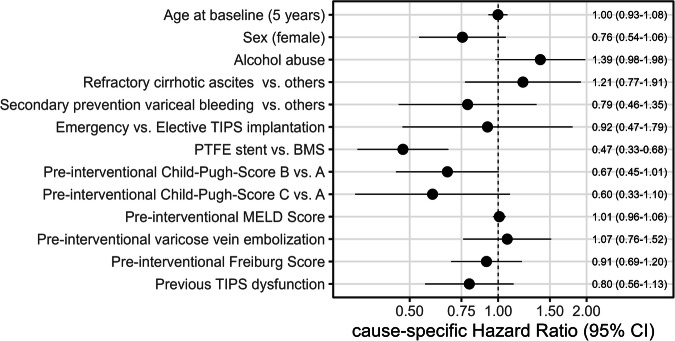
Cause-specific hazard ratio estimates along with 95% confidence intervals (CI) derived from a multivariable competing risk frailty model to identify factors independently associated with the instantaneous risk of TIPS dysfunction. The event of interest was dysfunction. BMS—bare metal stent (uncovered stent). PTFE stent—polytetrafluoroethylene stent (covered stent)

Using a competing risk frailty model, we found that patients with covered stents clearly had lower hazards for dysfunction compared to uncovered stents (HR 0.47, CI 0.33–0.68). Females had 24% lower hazards for dysfunction (HR 0.76, CI 0.54–1.06), just as patients with previous thrombosis (HR 0.80, CI 0.56–1.13), whereas age at TIPS did not show any association with dysfunction (HR 1.00, CI 0.93–1.08). CPS B or C were protective compared to score A, whereas the MELD score did not have a relevant independent association (HR 1.01, CI 0.96–1.06). Patients with alcohol abuse as the underlying disease had somewhat higher cause-specific hazards for dysfunction compared to others (HR 1.39, CI 0.98–1.98), independent of the other variables in the model. TIPS implantation for refractory cirrhotic ascites might be associated with dysfunction compared to other indications (HR 1.21, CI 0.77–1.91), while the TIPS indication secondary prevention of variceal bleeding had lower hazards (HR 0.79, CI 0.46–1.35). Elective TIPS implantation had slightly higher hazards for TIPS dysfunction compared to emergency implantation (HR 0.92, CI 0.47–1.97). The Freiburg score (0.91, CI 0.69–1.20) and pre-interventional vein embolization (HR 1.07, CI 0.76–1.52) had only a small effect (Fig. 3).

1-, 2-, and 5-year mortality rates were 20.9% (CI 17.7–24.1), 22.5% (CI 19.1–25.8), and 25.0% (CI 21.1–28.8), respectively (see Fig. 1. Overall, 23.6% of patients (*n* = 197) died during the follow-up period. Patients who died during follow-up were more often male (73.6% vs. 26.4%), had higher CPS (CPS C: 31.7% vs. 14.2%), and had higher pre-interventional MELD values (median of 17 vs. 13). The percentage of critically ill, bedbound patients was also higher among those who died during post-interventional follow-up (44.2% vs. 11.4%). Those who died during follow-up had elevated baseline bilirubin levels (2.13 mg/dL vs. 1.21 mg/dL) and lower platelet counts (103.5/nl vs. 131.0/nl) and showed higher rates of peri- and post-interventional complications (50.8% vs. 35.8%). The proportion of patients who died was higher for emergency TIPS interventions compared to elective implantations. In the post-interventional follow-up, liver transplant recipients had a higher mortality rate (6.1% vs. 2.4%) (Table 1).

**Fig. 4:**
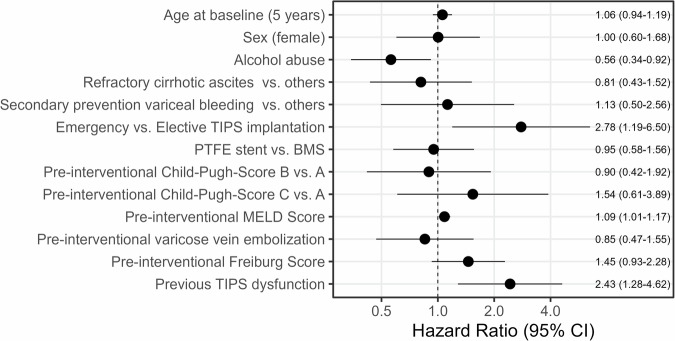
Cause-specific hazard ratio estimates along with 95% confidence intervals (CI) derived from a multivariable Cox regression model to identify factors independently associated with the instantaneous risk of post-interventional death. BMS—bare metal stent (uncovered stent). PTFE stent—polytetrafluoroethylene stent (covered stent)

A previous dysfunction increased the instantaneous risk of death 2.43-fold (HR 2.43, CI 1.28–4.62). Higher Freiburg and MELD score values were risk factors—given a 1-point increase in the Freiburg score, the hazards for death were 1.45-fold (HR 1.45, CI 0.93–2.28) and 1.09-fold for the MELD score (HR 1.09, CI 1.01–1.17). Sex and age at TIPS implantation had no or negligible effects on death (HR 1.00, CI 0.60–1.68) and HR 1.06, CI 0.94–1.19), just as the stent type (HR 0.95, CI 0.58–1.56). The CPS had no clear association with the hazards of death (Fig. 4). Patients with alcohol abuse as the underlying disease had lower hazards for death compared to others (HR 0.56, CI 0.34–0.92). The TIPS indication refractory ascites had slightly lower hazards for death compared to others (HR 0.81, CI 0.43–1.52), whereas the indication secondary prevention of variceal bleeding had slightly higher hazards for death than others (HR 1.13, CI 0.50–2.56). Pre-interventional varicose vein embolization was associated with lower hazards for death (HR 0.85, CI 0.47–1.55).

## Discussion

The aim of this study was to investigate dysfunction and mortality rates after TIPS procedures and to identify factors associated with these outcomes.

In our patient population, there was no relevant effect of patient age on the risk of TIPS dysfunction. Yet patient age was identified as a risk factor for TIPS dysfunction in other studies [22]. The female sex seems protective for the occurrence of dysfunction, but the data set is skewed regarding sex distribution, as the majority of treated patients are male. Yet female patients may be more compliant regarding, e.g., follow-up appointments, and also the rate of alcohol consumption was lower in this patient group which may account for the reduced risk of dysfunction. Patients with alcohol abuse as an underlying disease had a higher risk of developing TIPS dysfunction (HR 1.39). Published data for comparison do not exist. On the other hand, improved quality of life after TIPS (including a reduction in ascitic accumulation or variceal bleeding episodes) may lead to continued alcohol abuse and poorer compliance for follow-up appointments to ensure early detection of stent thrombosis. Recurrent alcohol use is also often seen in patients after liver transplantation [23]. Patients who underwent TIPS implantation because of refractory cirrhotic ascites also had a higher risk of TIPS dysfunction (HR 1.21) compared to other indications such as secondary prevention of variceal bleeding (HR 0.79). This may be due to more severe liver damage than those with variceal bleeding [24]. Yet, a higher CPS seemed to have a protective effect (CPS B HR 0.67 and CPS C HR 0.60), which may be attributable to impaired coagulation in advanced liver dysfunction since those patients also have a higher bleeding risk [25]. However, there are also studies showing that TIPS may even be detrimental in patients with refractory ascites and very advanced cirrhosis, such as CPS C patients [26]. In our analysis and independent of the other variables considered, a 1-unit increase in the MELD score had only a small effect (HR 1.01) regarding the development of thrombosis. Nevertheless, other investigators emphasize the significance of the MELD score for patient selection [27]. In particular, a MELD score > 18 was identified in other studies as a prognostic factor for dysfunction after TIPS implantation [28]. The Freiburg score had no relevant association with the occurrence of TIPS dysfunctions, but it must be noted that the score was developed to predict survival after TIPS [12].

For TIPS dysfunction due to thrombosis or stenosis, rates of 39-50% are reported to occur within the first year of TIPS placement [11], with even higher rates of up to about 80% when uncovered stents are used [26]. In our analysis, we also found higher thrombosis rates when uncovered stents were placed. Several published studies report better patency rates for covered stents (e.g., Sommer et al: 62% vs. 44%; Barrio et al: 100% vs. 18% [1]). While covered stents had a protective value regarding dysfunction (HR 0.47) in our analysis, the higher cost of covered stents may be a reason why uncovered stents are still frequently used [29]. Besides longer patency rates, covered stents also show better sealing to bile ducts and prevention of leakage or fistula formation [1]. It is surprising that an occurrence of thrombosis should be protective regarding further dysfunction; this may be explained by the better compliance of these patients and by an intensified follow-up. It is also possible that anticoagulation after re-interventions improves subsequent stent patency. Pre-interventional vein embolization had a negligible independent association with TIPS dysfunction, yet other studies show a higher 6-month overall rate of shunt patency in patients where TIPS implantation was combined with vein embolization [30, 31]. Other risk factors for TIPS dysfunction reported in the literature are a low body mass index (< 30 kg/m^2^) and TIPS procedures with no clinically evident success [22]. These factors could not be analyzed in our population. Experienced interventionalists (performing > 20 TIPS implantations per year) were identified as a possible protective factor for TIPS dysfunction in a retrospective study [32]. The experience of individual interventionalists could not be adequately represented in this study due to its retrospective design; yet with more than 32 interventions per year and an increasing volume of interventions our hospital has the status of a high-volume national reference center for TIPS implantation.

In our analysis, the cumulative 1-year mortality rate was 20.9%, which is lower than the 1-year mortality rate of 23% to 40% found in other retrospective studies [10, 11, 33]. It should be noted, however, that survival rates are also highly dependent on the indication for TIPS implantation with patients with refractory ascites having more advanced liver damage than those with variceal bleeding [24]. Indications have also changed since the introduction of TIPS 30 years ago [12]. While in the early years, the majority of TIPS procedures were performed for secondary prophylaxis of variceal hemorrhage, TIPS is now predominantly performed to treat refractory ascites [21]. Yet the TIPS indication refractory ascites in our patient cohort had slightly lower hazards for death (HR 0.81), whereas the indication secondary prevention of variceal bleeding had slightly higher hazards for death (HR 1.13). Other studies found contradictory data, although the reason for the difference in survival rates remains unclear [34].

Patient age and sex had no independent influence on survival. Nonetheless, there is evidence in the literature that mortality increases with age [35]. Surprisingly, patients with alcohol dependence showed higher survival rates than patients with other underlying conditions. Data in the literature are inconsistent, some studies indicate no effect on survival while other studies estimate alcoholic etiology as a risk factor for mortality [12, 35]. However, the limited compliance of patients with alcohol dependency could also contribute to the fact that deaths in this patient group were not recorded, as the patients were not in further contact with our clinic. The MELD score had a rather small independent effect (HR 1.09). Yet, the MELD score is a known prognostic parameter for mortality after elective TIPS [36]. A MELD score > 18 was identified as the cutoff in several studies [28, 33]. Patients with CPS C disease also had higher mortality rates than patients with CPS A (HR 1.54) in our analysis. Other studies found a threshold of > 11 (corresponding to CPS C) for post-interventional survival in high-risk patients [37]. However, it should be noted that the CPS has a distinctly subjective component (ascites, hepatic encephalopathy (HE)) and has limited validity for TIPS patients, as ascites and HE are significantly affected by the shunt itself [12]. Although both the MELD score, originally developed in the USA to estimate the prognosis after elective TIPS, and the Child-Pugh score, which is used to classify liver cirrhosis, have their undisputed value, they also have limitations, which is why the Freiburg index of post-TIPS survival (FIPS) has been proposed [12, 36]. The newly introduced FIPS was specifically developed as an alternative prognostic model for accurate survival prediction after TIPS implantation by Bettinger et al It includes age, bilirubin, albumin, and creatinine levels as prognostic factors [12]. In our analysis, the median FIPS score was 0.42 in the group of patients who died during follow-up. According to data from Bettinger et al, these patients have higher mortality rates at 3 months (around 20%) and 6 months (around 30%). In the group of patients who did not die, the FIPS score was lower (−0.17, 16.7% 3-month mortality rate, 19.4% 6-month mortality rate) [12]. Overall mortality in our study was comparable to the results of Bettinger et al with 16.6% at 3 months and 19.2% at 6 months. However, high FIPS scores seem to be a risk factor for shorter post-interventional survival (HR 2.43), confirming the results of Bettinger et al (14). Note though that published data on the validation of the FIPS score are not consistent. For example, in a large study population from North America, the performance of the FIPS score was comparable to that of MELD-Na in the era of modern TIPS treatment (2014–2020). In addition, further scientific data regarding model calibration are needed [38]. In our study population, emergency TIPS implantation was a risk factor regarding the post-interventional follow-up (HR 2.78). In an emergency setting, optimal preparation of the procedure is not always possible. Poor clinical outcomes after emergency TIPS implantation have also been described for patients with advanced liver disease [39, 40]. However, it must be considered that patients undergoing TIPS implantation due to acute variceal hemorrhage are a clinically different group than patients undergoing planned TIPS implantation. Yet the concept of “early-TIPS” within 72 h after acute variceal hemorrhage has become established for high-risk patients (age < 75 years, Child B or Child C ≤ 13 points, no HCC or HCC within Milan criteria, preserved cardiac and renal function) in recent years [41]. Patients with pre-interventional varicose vein embolization in this study had lower hazards for death (HR 0.85), yet other studies show similar rates of survival for TIPS alone and combined therapy with embolization [37].

Previous TIPS dysfunction was also associated with a higher risk of dying (HR 2.43) in our study population and has been reported as a risk factor for mortality in the literature [42, 43]. Controlling for previous dysfunction, uncovered stents were not relevantly associated with mortality (HR 0.95), which could well explain that uncovered stents increase the risk of dysfunction, which in turn increases the risk of mortality. Perarnau et al did not observe any significant difference with regard to HE or death between covered and uncovered stents [44]. Yet several studies suggest that covered stents are associated with better shunt patency, lower rates of clinical recurrence, and higher survival rates compared with uncoated stents [8, 45, 46]. Maleux et al reported that, in patients with refractory ascites, covered stents offered better symptomatic control of the ascites at 1-year follow-up and better overall survival [47]. Tan et al identified younger age and complete treatment as additional factors that contributed to better survival in patients treated with covered stents [42]. The recently published study by Wong et al showed that covered stents with a smaller diameter might contribute to a better quality of life and improved survival in carefully selected carefully selected patients with reasonable liver reserve and refractory ascites [48]. In addition, García-Pagán et al reported a survival benefit for the use of covered stents in patients with acute variceal bleeding, who are at a high risk of treatment failure [49]. In this high-risk population, covered stents have been increasingly used since 2010. In conclusion, there is abundant scientific evidence indicating that covered stents offer significant advantages over uncovered stents. Therefore, it is to be hoped that the reimbursement by health insurance companies in Germany will increase, helping covered stents to become established as standard in our hospital [50]. A patient-independent risk factor influencing mortality is the number of TIPS implantations performed per year; studies indicate lower in-patient mortality rates for hospitals performing > 20 interventions per year [32].

Our retrospective study has several limitations. Some data were incomplete, and documentation standards changed over the long observation period of 25 years. Moreover, interventional standards including the indications for TIPS implantation as well as interventionalists’ skills are likely to have improved over nearly three decades of experience (as published before). For example, during the 25-year study period covered by our analysis, the most frequent indication for TIPS implantation shifted from secondary prevention of variceal hemorrhage in the early years to treatment of recurrent ascites. We also found that more severely ill patients became TIPS candidates, including an increased proportion of patients with Child-Pugh C cirrhosis. The proportion of patients after liver transplant also increased. A major improvement in TIPS treatment was brought about by the advent of covered stents. Polytetrafluoroethylene (PTFE)-covered stents were licensed in 2003 and have since been increasingly used for TIPS because of advantages demonstrated in numerous studies, including longer patency rates, better sealing to bile ducts, prevention of leakage or fistulas, and a survival benefit in patients with refractory and/or recurrent ascites [1]. In our patient population, the use of covered stents only started to increase in 2010, mainly because of the higher costs of covered devices and therefore a lack of reimbursement by health insurance companies. Yet, all these technical improvements, changes in patient selection as well a gain in experience are likely to have contributed to higher success rates of TIPS over the years and might also have influenced the occurrence of dysfunction and mortality. The long study period may also be considered a limiting factor regarding the validation of the FIPS score, since Chapin et al point out that the performance of the FIPS score is particularly dependent on the time period considered, as for instance Bettinger et al demonstrated superior discrimination in a sample of German patients who underwent TIPS between 2000 and 2018 [12, 38]. However, many patients were lost to follow-up, for several reasons. On the one hand, compliance of many patients, especially those with alcohol dependence, was considerably reduced. In addition, many patients from smaller hospitals were transferred to our university center only for the TIPS procedure, and follow-ups were performed at the referring hospital or on an outpatient basis.

## Conclusion

The use of covered stents is an important protective factor regarding TIPS dysfunction. Similarly, female patients seem to have a lower risk of dysfunction while alcohol consumption increases the risk of dysfunction. Regarding survival, we could confirm the newly established Freiburg score in a patient cohort spanning the past 25 years. Furthermore, TIPS dysfunctions as well as TIPS implantations in emergency settings increase post-interventional mortality. Awareness of risk factors, like the use of uncovered stents, male sex, or the TIPS implantation setting could improve the selection of patients who may benefit from TIPS implantation and clinical follow-up, especially regarding early detection of TIPS dysfunction.

## Data Availability

Data is available on request.

## Abbreviations

BMS: Bare metal stent
CI: Confidence interval
CPS: Child-Pugh-Score
DSA: Digital subtraction angiography
ECOG: Eastern Cooperative Oncology Group
HCC: Hepatocellular carcinoma
HE: Hepatic encephalopathy
HR: Hazard ratio
IQR: Interquartile range
MELD: Model for end-stage liver disease
PSPG: Portosystemic pressure gradient
PTFE: Polytetrafluoroethylene
TIPS: Transjugular intrahepatic portosystemic shunt
